# Reverse Phenotyping Maternal Cystic Kidney Disease by Diagnosis in a Newborn: Case Report and Literature Review on Neonatal Cystic Kidney Diseases

**DOI:** 10.15388/Amed.2021.28.2.5

**Published:** 2021-08-02

**Authors:** Dovilė Ruzgienė, Meda Sutkevičiūtė, Birutė Burnytė, Kristina Grigalionienė, Augustina Jankauskienė

**Affiliations:** Faculty of Medicine, Vilnius University, Lithuania Vilnius University Hospital Santaros Klinikos; Faculty of Medicine, Vilnius University, Lithuania Vilnius University Hospital Santaros Klinikos; Faculty of Medicine, Vilnius University, Lithuania Vilnius University Hospital Santaros Klinikos; Faculty of Medicine, Vilnius University, Lithuania Vilnius University Hospital Santaros Klinikos; Faculty of Medicine, Vilnius University, Lithuania Vilnius University Hospital Santaros Klinikos

**Keywords:** kidney cysts, HNF1B, diabetes, family history

## Abstract

**Summary. **Kidney cysts are the most common kidney lesion, while congenital kidney cysts are mostly found in pediatric population. Neonatal kidney cysts can develop due to fetal malformations, rare genetic disorders or can be acquired which is very rare. Kidney cysts may be the only isolated finding or be part of the overall phenotype. They can be asymptomatic, found by ultrasound accidentally or can manifest from mild to life-threatening symptoms. Therefore, early diagnosis is very important. Autosomal dominant polycystic kidney disease and autosomal recessive polycystic kidney disease are the most common causes of kidney cysts in the neonatal population. This review highlights the most common kidney cystic diseases during the neonatal period and a rare clinical case of HNF1B-associated disease.

## Abstract (lt)

**Motinos cistinės inkstų ligos fenotipo nustatymas remiantis naujagimio diagnoze: klinikinio atvejo aprašymas ir literatūros apžvalga apie naujagimių cistines inkstų ligas:**

**Santrauka. **Inkstų cistos yra dažniausiai nustatomas inkstų pažeidimas. Vaikų populiacijoje dažnesnės įgimtos inkstų cistos. Naujagimiams inkstų cistos gali išsivystyti dėl vaisiaus malformacijų, retų genetinių ligų, o labai retai gali būti įgytos. Inkstų cistos gali būti vienintelis pavienis radinys arba fenotipo dalis. Jos gali būti besimptomės, nustatytos atsitiktinio ultragarsinio tyrimo metu, arba pasireikšti nuo lengvų iki gyvybei pavojingų simptomų. Todėl ankstyva diagnozė yra labai svarbi. Naujagimių populiacijoje dažniausiai pasireiškia autosominė dominantinė policistinė inkstų liga ir autosominė recesyvinė policistinė inkstų liga. Šiame straipsnyje apžvelgiamos dažniausios naujagimystės laikotarpiu pasireiškiančios cistinės inkstų ligos ir pristatomas retas su *HNF1B* genu susijusios ligos klinikinis atvejis.

**Raktažodžiai**: inkstų cistos, *HNF1B*, diabetas, šeiminė anamnezė.

## Introduction

Kidney cysts are the most common kidney lesion affecting up to 5% of the total population [1]. Pediatric kidney cystic diseases include a variety of hereditary or nonhereditary conditions. In neonates, kidney cysts usually occur due to genetic background. Only a small proportion of neonates and children develop simple cysts or acquired cystic kidney disease. Neonatal cystic kidney diseases can significantly contribute to neonatal morbidity and mortality, therefore understanding the various types of kidney cystic diseases in this period is crucial for a pediatric nephrologist as it can help with the early diagnosis and proper treatment. A clinical diagnosis of neonate cystic kidney disease is based upon identification of characteristic clinical symptoms, family history, and laboratory and kidney ultrasound findings. Hereditary cystic diseases are confirmed by genetic testing. Autosomal dominant polycystic kidney disease (ADPKD) and autosomal recessive polycystic kidney disease (ARPKD) are the most common causes of kidney cysts in the neonatal population. Less common causes include glomerulocystic kidney disease, juvenile nephronophthisis, Bardet–Biedl, Beckwith– Wiedemann, Von Hippel–Lindau, Meckel–Gruber syndromes. Some nonhereditary forms of poly-cystic kidney diseases include multicystic dysplastic kidney, obstructive cystic dysplasia, simple kidney cysts or complex kidney cysts [2]. Most common causes of hereditary kidney cysts are ARPKD, ADPKD, nephronophthisis, HNF1B-associated disease, tuberous sclerosis [3]. In this review article we will shortly discuss the most common kidney cystic diseases of infancy – ADPKD and ARPKD – as well as present a clinical case of a rare multisystemic disorder containing kidney cysts – HNF1B-associated disease.

## Epidemiology

The frequency of kidney cystic diseases in the neonatal population depends on a particular disease. The incidence of ADPKD is 1:400–1000 cases while ARPKD occurs in 1:20,000 live births [2, 4, 5]. HNF1B-associated disease is a very rare disease, its frequency is 1-9:1000000 [6].

## Etiology and pathophysiology

Kidney cystic diseases like ADPKD and ARPKD are part of the group of ciliopathies – hereditary disorders caused by mutation or the absence of genes that alter the structure and function of cilia [3]. Up to 90% of ADPKD cases have a family history and other cases are due to *de novo *mutations. The disease develops due to a mutation in the *PKD1* gene in the short arm of chromosome 16 (16p13.3) or in the *PKD2* gene in the long arm of chromosome 4 (4q21.2) [5]. 85% of cases are due to mutations in the *PKD1* gene. These genes are responsible for the production of polycystin 1 and polycystin 2 proteins [2, 5]. Polycystin 1 and polycystin 2 are responsible for the function of calcium channels. Patients with *PKD1* mutations have earlier onset of symptoms and earlier age of end stage kidney disease (ESKD) than those with *PKD2* mutations. ARPKD develops by disruption of fibrocystin/polyductin protein production due to a biallelic mutations in *PKHD1* gene [5]. Fibrocystin/ polyductin is responsible for the development of primary cilia, kidney and gallbladder epithelial cells resulting in normal nephrogenesis but excessive growth of collecting tubules [2, 5]. HNF1B-associated disease is caused by a mutation in the gene coding for the transcription factor hepatocyte nuclear factor 1-beta (*HNF1B*) on chromosome 17q12, it is inherited in autosomal dominant trait but up to 50% of cases occur due to spontaneous *de novo* mutations. 

## Signs and symptoms

Hereditary cystic diseases can cause severe kidney and other organs dysfunction. The onset of symptoms and their severity depends on the disease. ARPKD usually presents during pregnancy or at the age of the newborn but there are cases when the symptoms are observed only in adolescence or adulthood [2, 7, 8]. In contrast, ADPKD clinical presentation usually occurs in adolescence or adulthood between 20 and 40 years of age but there are cases when the disease is diagnosed during the antenatal period or in the newborn. Oligohydramnios, symmetrically enlarged hyperechogenic kidneys and small or no visualized bladder can be detected during pregnancy [9]. Newborns with ARPKD develop respiratory distress and impaired kidney function after birth. The most common kidney manifestations include hypertension and chronic kidney disease resulting in ESKD within 1 year in most cases [2, 10]. Moreover, Potter’s face (low-set ears, short nose, deep eye creases, macrognathia) and a large palpable mass in the abdomen can be observed. ARPKD may also affect the liver by causing congenital hepatic fibrosis, port hypertension and rising cholangitis. The most common ADPKD kidney manifestations in infancy include massively enlarged hyperechogenic kidneys, hypertension, macro- or microhematuria but there can be no symptoms during childhood at all [2]. Clinical manifestations that involve nonrenal organs comprise cardiac abnormalities (mitral valve prolapse, aortic aneurysms), cysts in other organs (liver, pancreas) and cerebral aneurysms which are extremely rare before the age of 18. HNF1B-nephropathy is recognized to represent an autosomal dominant syndromic disorder comprising kidney injury (kidney cysts, glomerular tufts, primitive tubules, irregular collecting systems, oligomeganephronia, enlarged kidney pelvises, abnormal calyces, small, single or horseshoe kidney), diabetes mellitus (maturity onset diabetes of the young type 5 (MODY5)), elevated cholestatic liver enzymes, hyperuricemia, pancreatic and genital tract malformations (vaginal aplasia, rudimentary uterus, bicornuate uterus, cryptorchidism, epididymal cysts and atresia of the vas deferens) [11]. MODY is a type of monogenic diabetes mellitus in which multiple genetic variants may cause an alteration to the functioning of beta cells. MODY5 is characteristic for HNF1B-associated disease, it usually develops in adolescence, up to the age of 25 years. HNF1B is responsible for early development of many organs therefore there is no phenotype–genotype correlation of HNF1B-associated disease, it can manifest in a wide range of phenotypes in the same family [12].

## Diagnostics

Ultrasound is the primary technique for evaluating kidneys in prenatal and postnatal period. It is inexpensive and harmless therefore it can be used for establishing the diagnosis as well as for the follow-up [3]. On prenatal ultrasound attention should be paid if oligohydramnios and/or kidney hyper echogenicity is detected because cysts may be invisible in this period [13]. In postnatal period ultrasound allows a detailed visualization of kidney parenchyma and of number, size and location of the cysts. The differential diagnosis of cystic kidney diseases can be made based on these findings. ARPKD ultrasound include bilateral enlarged and diffusely hyperechogenic kidneys, poor corticomedullary differentiation, microcysts less than 1 cm in diameter (usually 1–-2 mm in size, “salt and pepper” view) [2, 5, 9, 13, 14]. ADPKD ultrasound reveals enlarged and hyperechogenic kidneys, poor corticomedullary differentiation and large (greater than 1 cm) round cysts [2]. In HNF1B cases ultrasound findings are variable. Cysts can be found in only one or both kidneys, can be kidney hypoplasia, dysplasia, agenesis or even normal kidney [13]. If cysts are present, they are usually small, arising from the kidney cortex and do not tend to multiply over time [15]. Kidney ultrasound should be performed within the first 4 weeks after birth in neonates prenatally diagnosed with a solitary cyst. If unilateral kidney cysts or hyperechogenic kidney are diagnosed prenatally then kidney ultrasound should be performed between 3 and 7 days of age after birth [13].

Family history is very important in the diagnosis of cystic kidney disease. Kidney cysts may be suspected to be hereditary in case of a positive family history. However, lack of a positive family history does not necessarily exclude a patient from having a genetic disorder presenting with kidney cysts.

Hereditary cystic diseases are confirmed by genetic testing. Recommendations to perform a genetic testing includes: early onset bilateral cystic kidney disease or unilateral cystic kidney disease in combination with extrarenal manifestations detected prenatally; neonates with 2 or more kidney cysts and/or increased bilateral kidney cortical echogenicity [13]. Kidney function as well as functions of other organs that might be affected should be tested when a diagnosis of kidney cystic disease is suspected.

## Management and care

Treatment could be divided into prenatal and postnatal. In the presence of oligohydramnios induced pulmonary hypoplasia and in preterm (up to 34 weeks of gestation) labor 2 doses of corticosteroids for pulmonary maturation are recommended [13]. Postnatal management depends on the severity of a disease and is mostly conservative and supportive: maintenance of daily fluid balance, correction of electrolytes, hypertension, metabolic acidosis, anemia, maintenance of respiratory function [2, 4, 5, 16-18]. Kidney replacement therapy (dialysis, kidney transplantation) is recommended for neonates and children diagnosed with life-threatening kidney failure due to cystic kidney disease [1, 2, 13]. A liver and kidney transplantation may be necessary for patients with ESKD, advanced chronic kidney disease, or those with portal hypertension that cannot be managed medically or those who have recurrent cholangitis or clinically significant heart failure [2, 4].

## Case study

**Figure 1.**
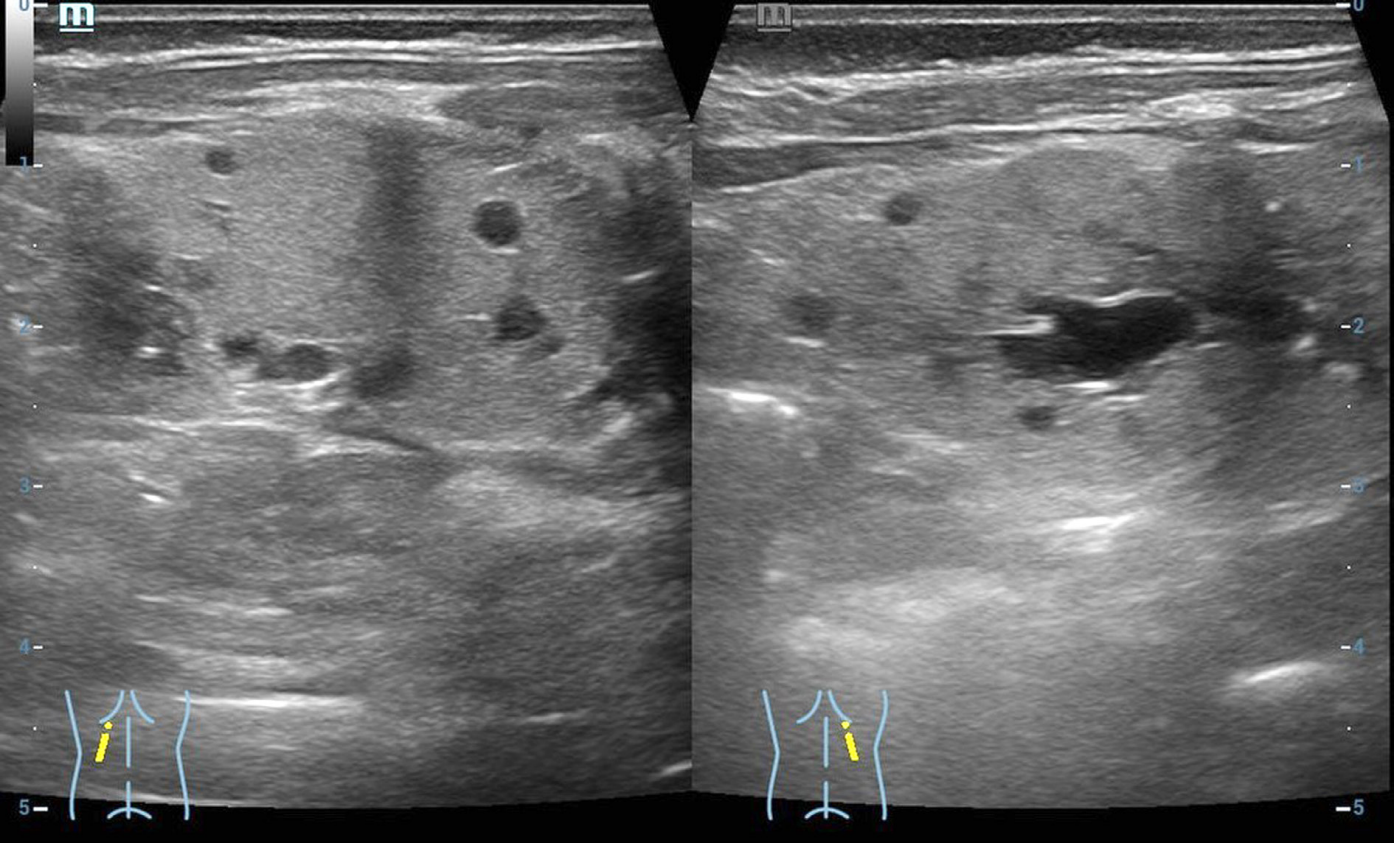
Patient kidney ultrasound. Increased echogenicity of the renal parenchyma on both sides, poor corticomedullary differentiation, thickened parenchyma, small multiple cysts in the parenchyma from immeasurable to 0.6 cm. The size of both kidneys 5 × 2.1 cm.

A baby girl was born after *in vitro* fertilization at 37 weeks of gestation (the labor was induced due to maternal preeclampsia) by vaginal delivery. At birth, her anthropometric measurements were as follows: weight 2320 g (<5th centile), height 50 cm (25th centile), and occipitofrontal circumference 33 cm (50th centile). Apgar scores were 8 and 9 at 1 and 5 min, respectively. Bilateral hyperechogenic kidneys were observed in fetus at 31^st^ week of gestation on prenatal ultrasound. Due to moaning infant was observed in the neonatal pathology ward for 9 hours. On the 3rd day of age, she developed a neonatal jaundice. Values for total bilirubin were elevated in capillary blood (438 µmol/L) and in venous blood (296 µmol/L). Laboratory testing showed metabolic acidosis (serum HCO3 17 mmol/L), hypercalcemia (ionized calcium 1.55 mmol/L). The neonate kidney ultrasound revealed poor corticomedullary differentiation, thickened parenchyma and small multiple cysts. No nephromegaly was observed (Figure 1). Renal function was impaired: increased creatinine (162 µmol/l) and reduced GFR (11 mL/min/1.73 m^2^). Urinalysis showed glucosuria (17 mmol/L), proteinuria (0.25 g/L), microhematuria (10/µl). The newborn was treated with a phototherapy and intravenous fluid infusion, sodium bicarbonate for the correction of metabolic acidosis. Total bilirubin went back to normal after two days of phototherapy, while metabolic acidosis was corrected after one week of treatment. The newborn was consulted by a pediatric nephrologist and clinical geneticist. Family history revealed: the mother was a 30-years old woman diagnosed with chronic kidney disease stage 3 (creatinine 122 µmol/L, GFR 51 ml/min/1.73 m^2^), diabetes mellitus at the age of 22, and had a 4-year history of unexplained primary infertility as well as hypertensive retinopathy. Her kidney ultrasound showed hypoplastic left kidney (left kidney 8.49 cm, parenchyma 1.13 cm; right kidney 10.86 cm, parenchyma 1.91 cm), bilateral kidney parenchymal cysts (1.35 to 2.29 cm in size), subcapsular microcalcinates, and reduced corticomedullary differentiation. At the age of 29 she was evaluated for infertility. Her gynecological examination results were normal, while laparoscopy and hysteroscopy findings included endometriosis and intrauterine polyp, respectively. Assisted reproduction attempt was performed without genetic counselling. At the time of pregnancy her BMI was 30.11 (class 1 obesity). Mild preeclampsia has been observed during pregnancy.

The newborn was clinically characterized as HNF1B-nephropathy based on a family history, objective examination and the results of laboratory and instrumental tests. On the 14th day of age, she was discharged home and continued to be followed up on an ambulatory care. After assessing maternal diseases and symptoms: chronic kidney disease (CKD), kidney cysts, gynecological problems, diabetes mellitus and in the presence of a newborn with suspected HNF1B nephropathy a genetic test was also performed for the mother.

**Figure 2.**
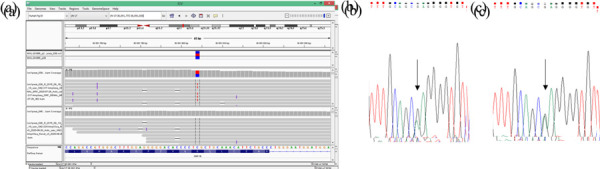
Detection of the pathogenic variant of *HNF1B* gene (NM_000458.4). a) Molecular analysis presenting variant c.827G>A (p.(Arg276Gln), CM060488 in *HNF1B* in a patient’s DNA from targeted NGS in Integrative Genomics Viewer (IGV). Sanger sequencing analysis revealed a presence of heterozygous *HNF1B* gene variant c.827G>A for the patient (b) and her mother (c). Arrow indicates variant site.

**Figure 3.**
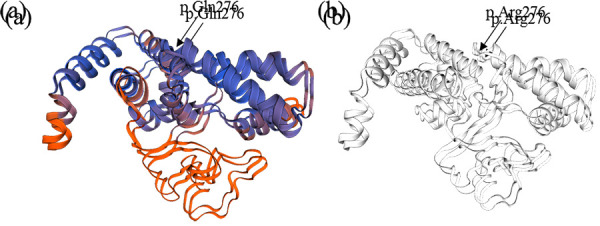
Hepatocyte nuclear factor 1-beta (HFN1B) protein structure homology model created using SWISS-MODEL web-based integrated service. A) Mutated protein with glutamine amino acid at 276 position. B) Wild type protein with arginine amino acid at 276 position.

A SNP-array analysis was performed in order to confirm the diagnosis. It did not detect any copy number variations in a 17q12.3 genomic region. The molecular diagnosis of HNF1B-associated disease by targeted next generation sequencing was obtained and confirmed by Sanger sequencing, which revealed variant was inherited from her mother. Sequence analysis identified a heterozygous pathogenic variant c.827G>A, (p.Arg276Gln) in *HNF1B*. (Figure 2 and 3).

## Discussion

The heterogenic presentation of HNF1B mutations can make the recognition of a disease very difficult in individual cases. Our patient was suspected to have an HNF1B-associated disease with kidney impairment based on family history, clinical, laboratory and ultrasound findings. Therefore the genetic testing was performed and diagnosis confirmed, moreover the diagnosis was established for the patient’s mother.

Many kidney and endocrine disorders might be explained by pathogenic variants of the *HNF1B* gene, the major transcription factor encoded in the 17q12.3 region or by deletions on this region. HNF1B is highly expressed in numerous fetal/adult tissues, where it mediates tissue specific gene expression, development and function [19]. Therefore, HNF1B-associated disease affects many organ systems and manifests a wide range of phenotypes, but there is a lack of genotype–phenotype correlation [20, 21]. HNF1B-associated disease is characterized by abnormalities in kidney structure and function, diabetes mellitus (MODY5 type), hyperuricemia, elevated liver cholestatic enzymes, pancreatic and genital tract malformations (synonym – renal cysts and diabetes syndrome, RCAD). No hyperuricemia, genital abnormalities, endocrine/exocrine insufficiency and elevated transaminases nor cholestatic enzymes were observed in our patient, but these signs may occur later in childhood. The disease is inherited in autosomal dominant trait but up to 50% of cases occur due to spontaneous *de novo* mutations so there is often no family history to make diagnose more quickly [5]. Interestingly, Okorn and colleagues have observed that a large proportion of familial cases are transmitted through the mother’s lineage, and this was previously observed by other group [21, 22]. In our case the mother was diagnosed with coexisting diseases: type 2 diabetes mellitus, chronic kidney disease, kidney cysts. All of these diseases together were the components of HNF1B-associated disease. After excluding deletion on 17q12.3 region, we performed targeted next generation sequencing analysis in affected newborn and identified the c.827G>A (p.(Arg276Gln) variant in the *HNF1B* gene. Subsequently, we performed segregation studies in the mother and confirmed the variant originated from her. Pathogenic *HNF1B* variant c.827G>A (p.(Arg276Gln) was first described in 2006 and reported in Human Gene Mutation Database (HGMD) [23]. The literature review indicates this pathogenic variant has been reported in four studies [23-26]. Two of these four cases were *de novo* and diagnosed prenatally [24, 25]. Bilateral hyperechogenic kidneys and cystic dysplasia are most commonly observed in prenatal *HNF1B* cases, followed by kidney cysts after birth [24, 27]. In our case kidney cysts were not detected on antenatal imaging, but bilateral hyperechogenic kidneys were observed on 31^st^ week of gestation. Ultrasound findings after birth are variable and nonspecific: cysts can be found in only one or both kidneys, can be kidney hypoplasia, dysplasia, agenesis or normal kidney [16]. Our newborn’s kidney ultrasound showed small up to 0.6 cm in size cysts in both kidneys parenchymas. Kidney cysts, oligomeganephronia, familial hyperuricemic nephropathy, kidney hypoplasia or dysplasia, multicystic dysplastic kidney, collecting system malformations, hypoplastic glomerulonephritic kidney disease and kidney failure are the most common postnatal kidney impairment features [5]. Patient was diagnosed with kidney cysts and kidney failure. Although the girl did not have all the criteria for Fanconi syndrome, the proximal tubular damage was observed: hyperkaliuria, hypercalciuria, glucosuria, proteinuria, hypermagnesuria, polyuria. Diabetes occurs between the ages of 10 and 40 years, usually before the age of 25 [20]. Our patient had normal glucose levels but her mother was diagnosed with type 2 diabetes at the age of 22 years, which is typical for the disease. Other rare manifestations reported in patients with HNF1B-associated disease include hypomagnesemia and hypocalciuria. [20, 21]. In contrast, in our case we diagnosed hypermagnesemia and hypercalciuria (urine calcium to creatinine ratio was 5.5). Hypermagnesemia was diagnosed at two months of age when the diagnosis of chronic kidney disease (CKD) was already established. The most common causes of hypermagnesemia in infants include: kidney impairment, parenteral magnesium infusion for the mother (used as treatment for pregnant woman with severe preeclampsia or eclampsia), high oral ingestion of magnesium [28, 29]. As our patient did not get any magnesium supplementation nor her mother was treated with magnesium sulfate, the most likely reason of hypermagnesemia in our case was the CKD. There are few mechanisms which lead to hypermagnesemia in CKD: low 1,25-dihydroxyvitamin D levels may decrease intestinal magnesium absorption, also hypermagnesemia develops when the GFR decreases to less than 30 ml/min/ m^2^ because magnesium excretion is decreased even though fractional excretion of magnesium is increased [28]. Abnormal tubular electrolyte processing associated with *HNF1B* pathogenic variants develops with age and is not limited to magnesium, but corresponds to a more generalized distal twisted tubular dysfunction [30]. Furthermore, in the case reported here, the mother was diagnosed with infertility. However, congenital malformations of the genital tract were not detected. To the best of our knowledge, although infertility has been reported in male patients with HNF1B-associated disease [31], it has not been previously reported in females with normal fertility evaluation. Nevertheless, it could still be a random feature unrelated to *HNF1B*.

Targeted NGS is a valuable and cost-effective method for the accurate diagnosis of various hereditary forms of the neonatal cystic kidney diseases. The identification of *HNF1B* pathogenic variant in the patient and her mother and the subsequent revision of maternal diagnosis from separated disorders to HNF1B-associated disease will have important implications for improved patient care, both in terms of treatment for the pre-existing phenotype, and also for long-term management of other HNF1B related features that may develop in the future. Long-term follow-up with regular pediatric nephrologist consultations are necessary for the child and appointments at nephrology department are necessary for the mother. The mother should be supervised by endocrinologist and dietitian as well. In case of further family planning the consultation of clinical geneticist should be performed.

## Conclusions

This case demonstrated the importance of a thorough family history in suspicion of the diagnosis of complex diseases with genetic etiologies and it may be helpful in alerting physicians to the rare association between kidney cysts and diabetes and need for genetic counselling. For families who carry identified pathogenic variants, assisted reproductive technology coupled with genetic diagnosis should be suggested. 
